# Direct Oral Anticoagulants Versus Warfarin in Atrial Fibrillation With Advanced Chronic Kidney Disease: A Systematic Review and Meta-Analysis

**DOI:** 10.7759/cureus.106043

**Published:** 2026-03-28

**Authors:** Ali S Metwaly, Aymen A Alqurain, Faisal F Alghanim, Nada A Alghamdi, Ragad K Alsudairi, Aref Y Dayl, Basil S Alghamdi, Osaid H Niazi, Fadel M Alfaraj, Nasser Q Alsharif, Amal Y Alfaifi, Mohammed A Alzahrani, Tala H Abdulmgid, Zainab A Alkaf

**Affiliations:** 1 Precision Medicine, Faculty of Pharmacy, Alexandria University, Alexandria, EGY; 2 Clinical Practice Department, Faculty of Pharmacy, Northern Border University, Rafha, SAU; 3 General Practice, King Saud Bin Abdulaziz University for Health Sciences, Riyadh, SAU; 4 Medical School, King Abdulaziz University Hospital, Jeddah, SAU; 5 Clinical Pharmacy, King Faisal University, Dammam, SAU; 6 Medical School, University of Science and Technology, Sana'a, YEM; 7 Medical School, College of Medicine, King Khalid University, Abha, SAU; 8 Medical School, University of Jeddah, Jeddah, SAU; 9 Internal Medicine, Saudi Commission for Health Specialties (SCFHS), Dammam, SAU; 10 Medical School, King Saud Bin Abdulaziz University for Health Sciences, Riyadh, SAU; 11 Pharmacy, Batterjee Medical College, Aseer Centre Hospital, Abha, SAU; 12 General Practice, College of Medicine, Cairo University, Giza, EGY; 13 Medical School, College of Medicine, Alexandria University, Alexandria, EGY; 14 Medical School, Ibn Sina National College for Medical Studies, Jeddah, SAU

**Keywords:** apixaban, atrial fibrillation, chronic kidney disease, dialysis, direct oral anticoagulants, end-stage renal disease, major bleeding, rivaroxaban, stroke prevention, warfarin

## Abstract

Patients with non-valvular atrial fibrillation (AF) and advanced chronic kidney disease (CKD), including those with end-stage kidney disease (ESKD) on dialysis, present a unique therapeutic challenge. This population is at an elevated risk for both thromboembolic events and severe bleeding complications. Because patients with creatinine clearance <25-30 mL/min were excluded from pivotal trials, the comparative safety and efficacy of direct oral anticoagulants (DOACs) versus traditional vitamin K antagonists (VKAs) in this cohort remain debated. A search of MEDLINE, Embase, and the Cochrane Central Register of Controlled Trials was performed from inception to the present to identify randomized controlled trials (RCTs) and adjusted observational studies comparing DOACs to VKAs in patients with AF and advanced CKD (stages 4-5 or dialysis). The primary outcomes were stroke or systemic embolism (SE) (efficacy) and major bleeding (safety). Hazard ratios (HRs) and 95% confidence intervals (CIs) were pooled using a random-effects (DerSimonian-Laird) model. Meta-regression, trial sequential analysis (TSA), and GRADE certainty assessments were applied. The final analysis included 21 studies (four RCTs, 17 observational cohorts) encompassing 184,136 participants. Compared to VKAs, DOACs were associated with a statistically significant 28% reduction in the risk of stroke or SE (heart rate (HR), 0.72; 95% confidence interval (CI), 0.60-0.86; *p* = 0.0004; moderate certainty). For safety, DOACs significantly reduced the risk of major bleeding by 26% compared to VKAs (HR, 0.74; 95% CI, 0.61-0.90; *p* = 0.0026; low to moderate certainty). However, substantial statistical heterogeneity was observed for the bleeding outcome (I^2^ = 80.2%). Heterogeneity was primarily driven by agent-specific effects, with apixaban demonstrating the most favorable safety profile. Meta-regression confirmed that the specific DOAC agent was a significant moderator of bleeding risk (*p* < 0.0001), with apixaban (HR, 0.63) and rivaroxaban (HR, 0.75) driving the safety benefits, whereas dabigatran was associated with increased bleeding (HR, 1.48). TSA for stroke reduction indicated that while the cumulative Z-curve crossed the conventional benefit boundary, the information size remained below the heterogeneity-adjusted requirement. In patients with AF and advanced CKD or ESKD requiring dialysis, the use of DOACs, specifically apixaban and rivaroxaban, demonstrated superior efficacy in stroke prevention and a safer bleeding profile compared to VKAs. These findings suggest that factor Xa inhibitors, specifically apixaban and rivaroxaban, should be preferred over VKAs in this high-risk population; however, adequately powered RCTs are needed to refine specific dosing strategies.

## Introduction and background

Atrial fibrillation (AF) and chronic kidney disease (CKD) represent a growing dual epidemic, sharing common risk factors and a bidirectional pathophysiological relationship that accelerates the progression of both conditions [1]. The prevalence of AF increases as renal function declines, affecting approximately 20-30% of patients with advanced CKD and up to 40% of those with end-stage kidney disease (ESKD) undergoing dialysis [2,3]. This specific population presents a unique therapeutic dilemma, as they face an elevated risk of thromboembolic events compared to the general population, but simultaneously harbour a distinct hemostatic profile characterized by uremic platelet dysfunction and altered vessel wall interaction, leading to a disproportionately high risk of major bleeding [4,5].

Vitamin K antagonists (VKAs), such as warfarin, are the mainstay of stroke prevention in patients with AF. However, their use in advanced CKD is fraught with challenges, including unpredictable pharmacokinetics, frequent supratherapeutic anticoagulation due to reduced cytochrome P450 activity, and difficulty maintaining the time in therapeutic range [2,6]. In addition, VKA therapy in this population has been linked to accelerated vascular calcification and the catastrophic complication of calciphylaxis [5,7]. Observational data and previous systematic reviews have yielded conflicting results regarding the net clinical benefit of VKAs in patients with ESRD, with some studies suggesting no reduction in ischemic stroke and a marked increase in hemorrhagic events compared to no anticoagulation [8,9].

Direct oral anticoagulants (DOACs), including dabigatran, rivaroxaban, apixaban, and edoxaban, have demonstrated non-inferiority or superiority to warfarin in the general AF population. However, patients with advanced CKD (creatinine clearance <25-30 mL/min) were systematically excluded from the pivotal randomized controlled trials (RCTs) establishing DOAC efficacy [10,11]. This exclusion was driven by concerns over drug accumulation, as all DOACs depend on renal clearance to varying degrees (approximately 27% for apixaban, 33% for rivaroxaban, 50% for edoxaban, and 80% for dabigatran) [5,10]. Guidelines for anticoagulation in patients with stage 4-5 CKD and those on dialysis remain disparate, often depending on extrapolated pharmacokinetic data or observational evidence rather than robust clinical endpoints [12].

Recent years have seen the emergence of dedicated RCTs and high-quality observational studies focusing on this high-risk cohort; however, the results remain inconsistent. Some meta-analyses suggest that DOACs, particularly apixaban and rivaroxaban, may offer a safer bleeding profile than VKAs without compromising thromboembolic protection [13,14], whereas others indicate no significant difference in all-cause mortality or stroke rates between treatment strategies [15,16]. Furthermore, the safety signal regarding specific agents remains debated, as apixaban is favoured due to lower renal elimination; however, evidence regarding the safety of other factor Xa inhibitors in advanced renal failure is less established [17,18]. In addition, recent analyses have highlighted that randomized trials in this specific population have often been underpowered or terminated early, requiring a rigorous synthesis of available evidence [19,20].

Because of the persistent clinical equipoise and the introduction of new data since previous reviews, an updated synthesis is required. This systematic review and meta-analysis aim to evaluate the comparative efficacy and safety of DOACs versus warfarin specifically in patients with non-valvular AF and advanced CKD (stage 4-5 and dialysis-dependent), providing a comprehensive assessment of ischemic stroke, major bleeding, and mortality outcomes to inform evidence-based clinical practice. Unlike prior reviews, this synthesis incorporates the most recently published RCTs and large-scale cohorts, utilizes trial sequential analysis (TSA) to evaluate the conclusiveness of cumulative evidence, and employs meta-regression to robustly isolate agent-specific effects on safety.

## Review

Methods

Protocol and Registration

This systematic review and meta-analysis was conducted in accordance with the Preferred Reporting Items for Systematic Reviews and Meta-Analyses (PRISMA) guidelines [21] and the Meta-analysis of Observational Studies in Epidemiology (MOOSE) guidelines for observational studies. The study protocol was prospectively registered with the International Prospective Register of Systematic Reviews (PROSPERO; CRD420261320626).

Data Sources and Search Strategy

A systematic search was performed on MEDLINE, Embase, and the Cochrane Central Register of Controlled Trials (CENTRAL) from inception to the present. The search strategy utilized a combination of Medical Subject Headings (MeSH) and free-text terms relevant to "atrial fibrillation", "chronic kidney disease", " end-stage renal disease", " dialysis", " warfarin", " vitamin K antagonists", and specific direct oral anticoagulants (DOACs: apixaban, rivaroxaban, dabigatran, edoxaban) (Table 1). The reference lists of eligible studies and relevant review articles were manually screened to identify additional citations.

**Table 1:** Search strategy

Database	Search terms (MeSH and Free-text combinations)
MEDLINE	"atrial fibrillation" AND ("chronic kidney disease" OR "end stage renal disease" OR "dialysis") AND ("warfarin" OR "vitamin K antagonists" OR "direct oral anticoagulants" OR "apixaban" OR "rivaroxaban" OR "dabigatran" OR "edoxaban")
Embase	"atrial fibrillation" AND ("chronic kidney disease" OR "end stage renal disease" OR "dialysis") AND ("warfarin" OR "vitamin K antagonists" OR "direct oral anticoagulants" OR "apixaban" OR "rivaroxaban" OR "dabigatran" OR "edoxaban")
Cochrane Central Register of Controlled Trials (CENTRAL)	"atrial fibrillation" AND ("chronic kidney disease" OR "end stage renal disease" OR "dialysis") AND ("warfarin" OR "vitamin K antagonists" OR "direct oral anticoagulants" OR "apixaban" OR "rivaroxaban" OR "dabigatran" OR "edoxaban")

Eligibility Criteria and Study Selection

Studies meeting the following criteria were included (Table 2): (1) population: adult patients with non-valvular atrial fibrillation and advanced CKD, defined as an estimated glomerular filtration rate (eGFR) <30 mL/min/1.73 m² or undergoing dialysis (hemodialysis or peritoneal dialysis); (2) intervention: treatment with a DOAC (apixaban, rivaroxaban, dabigatran, or edoxaban); (3) comparator: treatment with warfarin or other VKAs; (4) outcomes: quantitative reporting of efficacy (stroke/SE) or safety (major bleeding, intracranial hemorrhage, or gastrointestinal bleeding) outcomes; and (5) study design: RCTs and observational studies (prospective or retrospective cohorts) reporting adjusted hazard ratios (HRs). Two independent reviewers screened titles, abstracts, and full texts, and disagreements were resolved by a third reviewer. Inter-rater reliability during the screening process was quantified using Cohen’s kappa statistic (κ) [22].

**Table 2:** Inclusion and exclusion criteria

Criteria category	Inclusion criteria	Exclusion criteria
Population	Adult patients (≥18 years) with non-valvular atrial fibrillation and advanced CKD (eGFR <30 mL/min/1.73 m²) or undergoing dialysis (hemodialysis or peritoneal dialysis)	Patients with valvular atrial fibrillation, mild-to-moderate CKD (eGFR ≥30 mL/min/1.73 m²), or pediatric populations
Intervention	Treatment with a direct oral anticoagulant (DOAC: apixaban, rivaroxaban, dabigatran, or edoxaban)	Treatment with anticoagulants other than DOACs
Comparator	Treatment with warfarin or other vitamin K antagonists (VKAs)	No comparator group or comparator other than VKAs
Outcomes	Quantitative reporting of efficacy (stroke/systemic embolism) or safety (major bleeding, intracranial hemorrhage, or gastrointestinal bleeding)	Insufficient data to extract hazard ratios for relevant outcomes
Study design	Randomized controlled trials (RCTs) and observational studies (prospective or retrospective cohorts) reporting adjusted hazard ratios (HRs)	Case reports, case series, non-adjusted observational studies, and reviews; short follow-up duration (<30 days) or admission for acute illness only

Data Extraction and Quality Assessment

Data extraction was independently performed by two investigators using a standardized electronic data collection form. For observational studies, maximally adjusted HRs (e.g., those derived from propensity score matching or multivariate regression) were extracted to minimize confounding bias. When studies reported both, propensity score-matched hazard ratios were preferentially extracted over multivariate regression-adjusted estimates to maximize control for residual confounding. The methodological quality and risk of bias were assessed using design-specific tools: the Cochrane Risk of Bias 2 (RoB 2) tool [23] was employed for RCTs, evaluating domains such as randomization process, deviations from intended interventions, and missing outcome data. The Risk of Bias in Non-randomized Studies of Interventions (ROBINS-I) tool [24] was used for observational studies to assess bias across seven domains, including confounding and participant selection.

Statistical Analysis

All statistical analyses were performed using R (version 4.5.2; R Foundation for Statistical Computing, Vienna, Austria) [25].

Data Synthesis

The principal summary measure was the hazard ratio (HR) with 95% confidence intervals (CI). To account for anticipated clinical and methodological heterogeneity across the included RCTs and observational studies, log-transformed HRs were pooled using the generic inverse-variance method within a random-effects model (DerSimonian-Laird estimator) [26]. Prediction intervals were calculated to estimate the range within which the effect of a future study would be expected to fall.

Heterogeneity and Consistency

Statistical heterogeneity was evaluated using the Cochran Q test and quantified using the I2 statistic [27], where I2 values of 25%, 50%, and 75% represented low, moderate, and high heterogeneity, respectively. Between-study variance was further assessed using tau-squared (τ2) [28].

Publication Bias

Publication bias was assessed visually using funnel plots for outcomes involving more than 10 studies. Asymmetry was statistically evaluated using Egger’s linear regression test [29] and Begg’s rank correlation test [30].

Subgroup and Sensitivity Analyses

To assess the robustness of the results and explore sources of heterogeneity, pre-specified subgroup analyses and meta-regression were conducted based on (1) specific DOAC agent (apixaban vs. rivaroxaban vs. dabigatran), (2) CKD severity (dialysis-dependent vs. stage 4/5 non-dialysis), (3) dosing regimen (standard vs. reduced dose), and (4) study design (RCT vs. observational).

Trial Sequential Analysis

TSA was performed to evaluate the sufficiency of the current evidence and to control for type I and type II errors associated with repetitive testing of accumulating data. The required information size (RIS) was calculated to determine whether the cumulative Z-curve crossed the trial sequential monitoring boundaries for benefit, harm, or futility [31].

Certainty of Evidence

The certainty of the body of evidence for primary outcomes was appraised using the Grades of Recommendations Assessment, Development, and Evaluation (GRADE) approach [32], classifying evidence as high, moderate, low, or very low quality based on risk of bias, inconsistency, indirectness, imprecision, and publication bias.

Results

Study Selection and Inter-Rater Reliability

**Figure 1:**
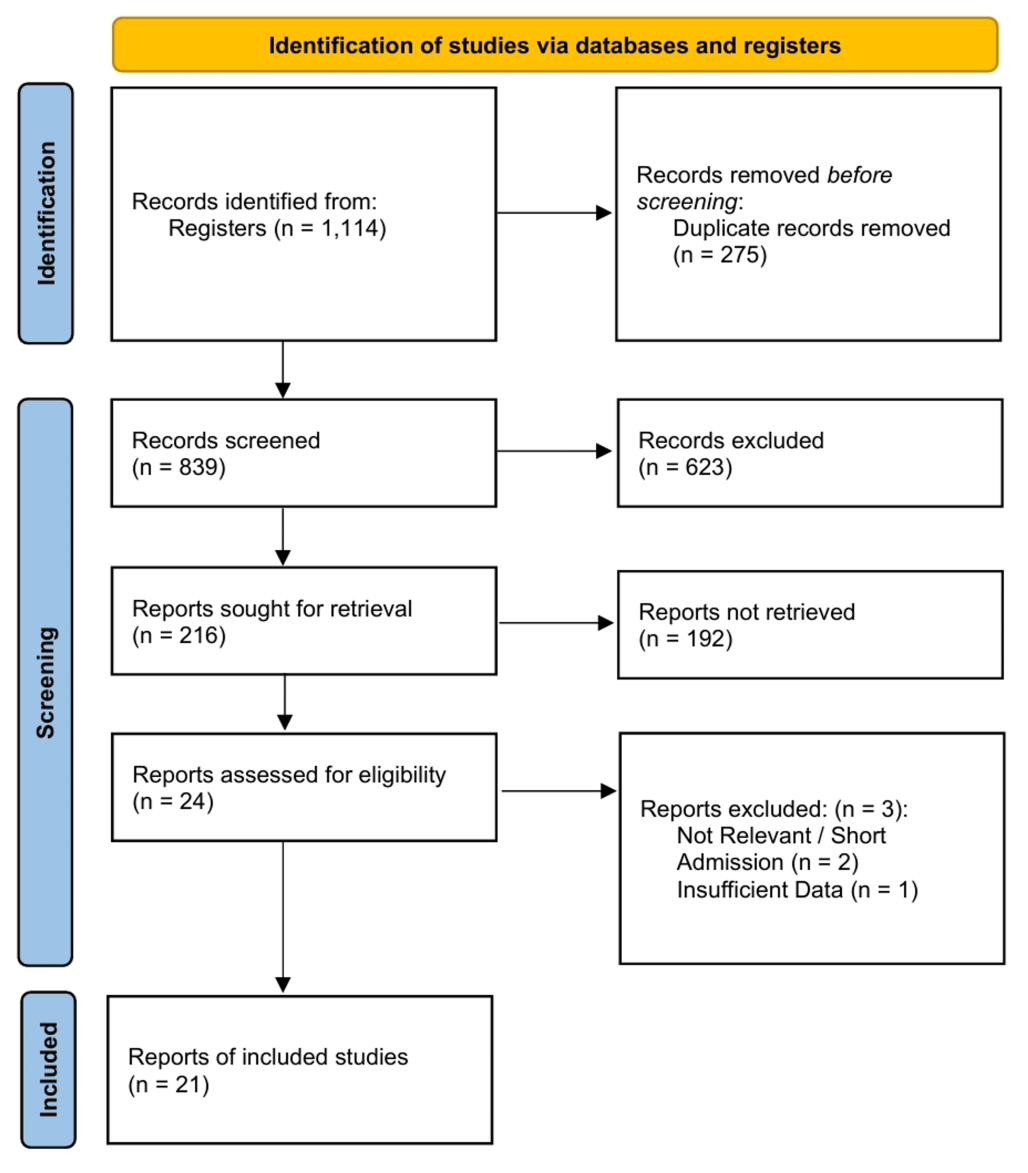
PRISMA 2020 flow diagram detailing the study selection process.

A systematic search of the literature identified 1,114 records from registers and databases. Following the removal of 275 duplicate records, 839 titles and abstracts were screened. Of these, 623 were excluded, leaving 216 reports sought for retrieval. After screening, 24 eligible articles were retrieved and underwent full-text assessment; of these, three were subsequently excluded (two for non-relevant or short admission criteria and one for insufficient data). Twenty-one studies [33-53] met all inclusion criteria and were included in the qualitative synthesis, with 18 contributing to the quantitative meta-analysis (Figure 1).

Agreement between the two independent reviewers during the study selection process was high. The calculated Cohen’s kappa statistic was 0.694 (z = 3.12, p = 0.0018), indicating a good level of inter-rater reliability.

Characteristics of the Included Studies

Twenty-one studies published between 2015 and 2024 in the systematic review, of which 18 provided sufficient adjusted HR data for quantitative meta-analysis. The final cohort comprised four RCTs [40-42,53] and 17 observational studies [33-39,43-52]. Geographically, the studies were conducted across the United States, Taiwan, Sweden, France, Germany, and Belgium, utilizing major national registries such as the USRDS, MarketScan, NHIRD, and the Swedish Renal Registry.

The study population consisted of adult patients with non-valvular AF and advanced renal impairment, primarily ESKD on maintenance hemodialysis or stage 4-5 CKD. A total of 184, 136 participants were analysed across the pooled outcomes. Baseline stroke risk was high, with mean or median CHA2-DS2-VASc scores ranging from 3.0 to 6.1. Similarly, baseline hemorrhagic risk was elevated, with HAS-BLED scores typically ≥3.0. Apixaban was the most frequently investigated DOAC agent, followed by rivaroxaban and dabigatran. Warfarin was the near-universal comparator, although phenprocoumon was utilized in the German and Belgian trials [41,42]. Follow-up periods ranged from one to seven years, reflecting both short-term surgical outcomes and long-term anticoagulation management (Table 3).

**Table 3:** Baseline characteristics of the included studies Abbreviations: AF, atrial fibrillation; Api, apixaban; BID, twice daily; CKD, chronic kidney disease; CrCl, creatinine clearance; Dabi, dabigatran; DOAC, direct oral anticoagulant; Edo, edoxaban; eGFR, estimated glomerular filtration rate; ESKD, end-stage kidney disease; HD, hemodialysis; NHIRD, National Health Insurance Research Database; PD, peritoneal dialysis; QD, once daily; RCT, randomized controlled trial; Riva, rivaroxaban; SRR, Swedish Renal Registry; USRDS, United States Renal Data System; VKA, vitamin K antagonist (warfarin/phenprocoumon) De Vriese et al. included two rivaroxaban arms (with and without vitamin K2); these were pooled for the DOAC comparison in this analysis. † Chan et al. also reported on rivaroxaban and warfarin; dabigatran data are highlighted here as it is the primary source for dabigatran safety in dialysis. ‡ Welander et al. 2023 sample size represents the G5/dialysis subgroup extracted for subgroup analysis.

Study ID	Study design and data source	Population characteristics	Total N (DOAC / VKA)	Intervention (DOAC)	Patient demographics (mean/median age; % male)	Renal status / inclusion criteria
Randomized controlled trials (RCTs)						
Pokorney et al., 2022 [40]	RCT (RENAL-AF), USA	ESKD on hemodialysis; AF	154 (82 / 72)	Apixaban (5 mg or 2.5 mg BID)	69 years, 63.6% male	Hemodialysis
Reinecke et al., 2023 [41]	RCT (AXADIA-AFNET 8), Germany	ESKD on hemodialysis; AF	97 (48 / 49)	Apixaban (2.5 mg BID)	75 years, 70.1% male	Hemodialysis
De Vriese et al., 2021 [42]	RCT (VALKYRIE), Belgium	ESKD on hemodialysis; AF	132 (88 / 44)*	Rivaroxaban (10 mg QD)	80 years, 56.8% male	Hemodialysis
Stanifer et al., 2020 [53]	RCT (Post-hoc ARISTOTLE), Global	AF with CrCl 25–30 mL/min	269 (136 / 133)	Apixaban (5 mg or 2.5 mg BID)	81 years, 39.4% male	CrCl 25–30 mL/min
Observational studies						
Fu et al., 2024 [34]	Retrospective cohort, USA (Medicare/Optum)	Advanced CKD (Stage 4–5); AF	24,976 (12,488 / 12,488)	Apixaban (mixed doses)	79 years, 49.6% male	CKD Stage 4–5 (non-dialysis)
Siontis et al., 2018 [43]	Retrospective cohort, USA (USRDS)	ESKD on dialysis; AF	9,404 (2,351 / 7,053)	Apixaban (mixed doses)	68 years, 54.4% male	Dialysis (HD/PD)
Wetmore et al., 2022 [44]	Retrospective cohort, USA (USRDS)	ESKD on dialysis; AF	17,156 (4,639 / 12,517)	Apixaban (5 mg or 2.5 mg BID)	66 years, 61.7% male	Dialysis (HD/PD)
Laville et al., 2024 [50]	Retrospective cohort, France (REIN registry)	ESKD on dialysis; AF	8,954 (483 / 8,471)	Mixed DOACs (Api, Riva, Dabi)	73 years, 63% male	Dialysis
Coleman et al., 2019 [45]	Retrospective cohort, USA (MarketScan)	Stage 4–5 CKD or dialysis; AF	6,744 (1,896 / 4,848)	Rivaroxaban (mixed doses)	72 years, 58% male	CKD 4–5 or Dialysis
Weir et al., 2020 [39]	Retrospective cohort, USA (Optum)	Stage 4–5 CKD; AF	2,317 (781 / 1,536)	Rivaroxaban (mixed doses)	80 years, 39.5% male	CKD 4–5 (non-dialysis)
See et al., 2021 [33]	Retrospective cohort, Taiwan (NHIRD)	ESKD on dialysis; AF	3,237 (490 / 2,747)	Mixed DOACs (Api, Riva, Dabi)	74 years, 49.6% male	Dialysis
Hsu et al., 2023 [35]	Retrospective cohort, Taiwan (TPEVGH)	Advanced CKD (eGFR <30); AF	1,011 (809 / 202)	Mixed DOACs (Api, Riva, Dabi, Edo)	84 years, 57.3% male	eGFR <30 mL/min
Lin et al., 2021 [47]	Retrospective cohort, Taiwan (NHIRD)	ESKD on dialysis; AF	3,358 (173 / 3,185)	Rivaroxaban (mixed doses)	75 years, 55% male	Dialysis
Chan et al., 2015 [46]	Retrospective cohort, USA (Fresenius)	ESKD on hemodialysis; AF	8,345 (281 / 8,064)†	Dabigatran (mixed doses)	68 years, 59% male	Hemodialysis
Welander et al., 2023 [36]	Retrospective cohort, Sweden (SRR)	CKD G3–G5D; AF	1,172 (143 / 1,029)‡	Mixed DOACs (Api, Riva, Dabi, Edo)	77 years, 68% male	Stage 5 / Dialysis subset
Welander et al., 2024 [37]	Retrospective cohort, Sweden (SRR)	CKD G3–G5D; AF	2,453 (1,005 / 1,448)	Mixed DOACs	77 years, 68% male	Stage 3–5D (Mixed)
Schafer et al., 2018 [38]	Retrospective cohort, USA (single center)	Stage 4–5 CKD or dialysis; AF	604 (302 / 302)	Apixaban (mixed doses)	73 years, 46% male	CKD 4–5 or Dialysis
Ionescu et al., 2021 [52]	Retrospective cohort, USA (multi-center)	ESKD on hemodialysis; AF	707 (144 / 563)	Apixaban (5 mg or 2.5 mg BID)	67 years, 59% male	Hemodialysis
Sarratt et al., 2017 [48]	Retrospective cohort, USA (single center)	ESKD on hemodialysis; AF	160 (40 / 120)	Apixaban (mixed doses)	71 years, 50% male	Hemodialysis
Stanton et al., 2017 [49]	Retrospective cohort, USA (single center)	Severe renal impairment; AF	146 (73 / 73)	Apixaban (mixed doses)	79 years, 40% male	CrCl <25 or SCr >2.5
Moore et al., 2024 [51]	Retrospective cohort, USA (single center)	ESKD on hemodialysis; AF	110 (53 / 57)	Apixaban (mixed doses)	69 years, 55% male	Hemodialysis

Risk-of-Bias Assessment

**Figure 2:**
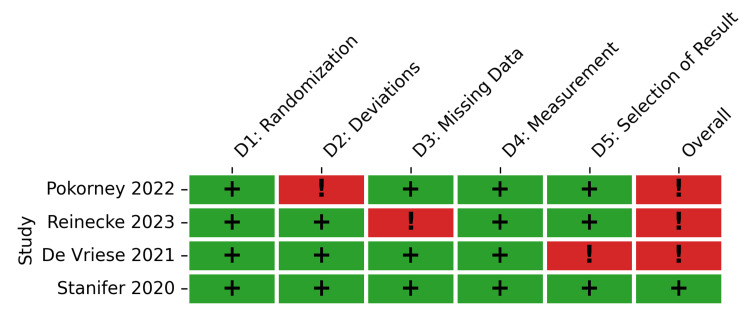
Risk of bias 2 (RoB 2) traffic light plot for the included randomized controlled trials. Study citations: Pokorney et al. 2022 [40], Reinecke et al. 2023 [41], De Vriese et al. 2021 [42], and Stanifer et al. 2020 [53]

**Figure 3:**
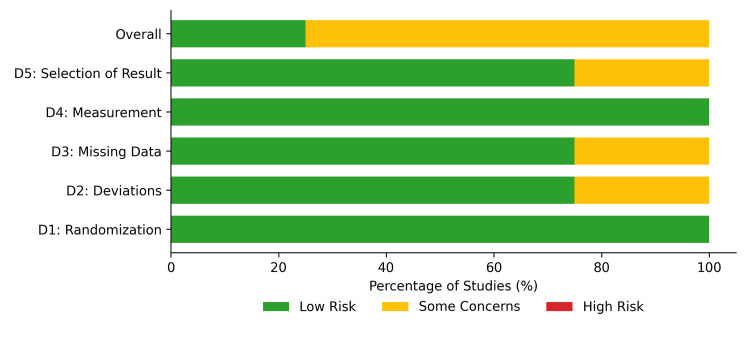
Risk of bias 2 (RoB 2) summary. Summary plot representing the proportion of randomized controlled trials within each bias domain.

The methodological quality of the included studies varied by study design. The RoB 2 tool demonstrated generally low risk across most domains for the four included RCTs. Randomization (D1), measurement of outcomes (D4), and selection of reported results (D5) were assessed as low risk in 75-100% of the RCTs. However, concerns regarding deviations from intended interventions (D2) and missing outcome data (D3) were noted, resulting in some concerns (moderate risk) for the overall risk of bias in three of the four trials, with only one RCT evaluated as having a low overall risk of bias (Figures 2, 3).

**Figure 4:**
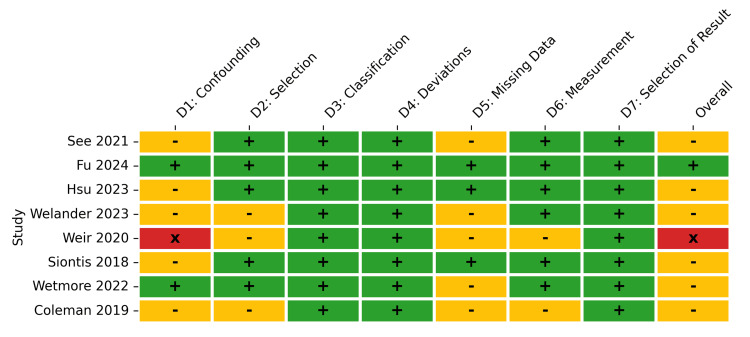
ROBINS-I traffic light plot for included observational studies. Study citations: See et al. 2021 [33], Fu et al. 2024 [34], Hsu et al. 2023 [35], Welander et al. 2023 [36], Weir et al. 2020 [39], Siontis et al. 2018 [43], Wetmore et al. 2022 [44], Coleman et al. 2019 [45] A "moderate" risk of bias for confounding indicates that the study adjusted for key prognostic variables (e.g., via propensity score matching or multivariate regression), but residual confounding cannot be entirely ruled out. A "serious" risk indicates that important domains of confounding were not appropriately controlled for.

**Figure 5:**
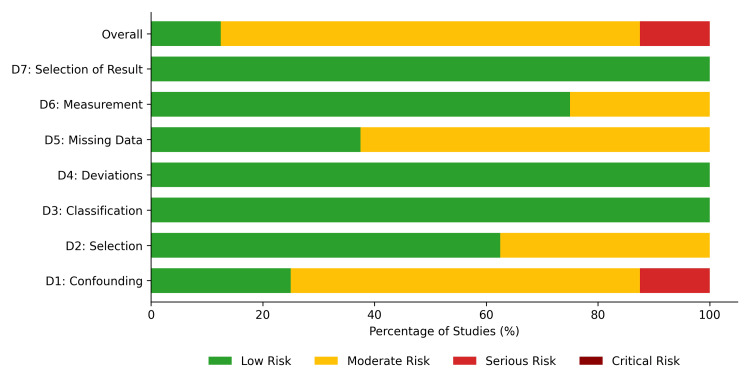
ROBINS-I summary. Summary plot representing the proportion of observational studies within each bias domain. A "moderate" risk of bias for confounding indicates that the study adjusted for key prognostic variables (e.g., via propensity score matching or multivariate regression), but residual confounding cannot be entirely ruled out. A "serious" risk indicates that important domains of confounding were not appropriately controlled for.

For the observational studies, the ROBINS-I assessment revealed a moderate risk of bias. While domains such as classification of interventions (D3) and deviations from intended interventions (D4) were uniformly rated as low risk across all studies, the domain for confounding (D1) presented the most significant source of potential bias. Specifically, 62.5% of observational studies were rated at moderate risk for confounding, 25% at low risk, and 12.5% at serious risk. Consequently, the overall risk of bias for the observational cohort was rated as moderate for the majority (75%) of the included studies, low for 12.5%, and serious for 12.5% (Figures 4, 5).

Primary Efficacy Outcome: Stroke or SE

**Figure 6:**
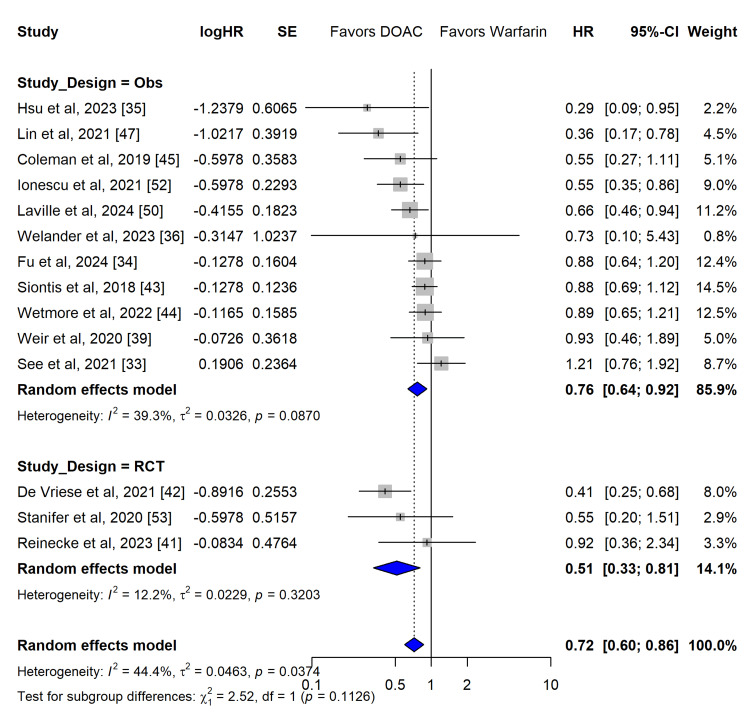
Primary efficacy outcome (stroke or systemic embolism) Forest plot for the primary efficacy outcome, including subgroup analysis by study design (randomized controlled trial (RCT) vs. observational) and calculated prediction interval.

Data for the primary efficacy endpoint - stroke or SE - were pooled from 14 studies encompassing both RCT and observational designs. In the random-effects meta-analysis, the use of DOACs was associated with a statistically significant 28% relative risk reduction (RRR) in stroke/SE compared to warfarin (hazard ratio (HR), 0.72; 95% confidence interval (CI), 0.60-0.86; z = -3.53; p = 0.0004) (Figure 6).

Moderate statistical heterogeneity was observed across the efficacy estimates (I_2_ = 44.4%, 95% CI: 0.0-70.3%; Cochran’s Q = 23.37, p = 0.0374; τ2 = 0.0463). To account for this between-study variance, a prediction interval was calculated, yielding an interval of 0.43 to 1.19. This wide prediction interval, which crosses the line of unity (1.0), suggests that while the mean effect strongly favours DOACs, the true effect in a future individual clinical setting may occasionally favour warfarin or demonstrate no difference.

Primary Safety Outcome: Major Bleeding

**Figure 7:**
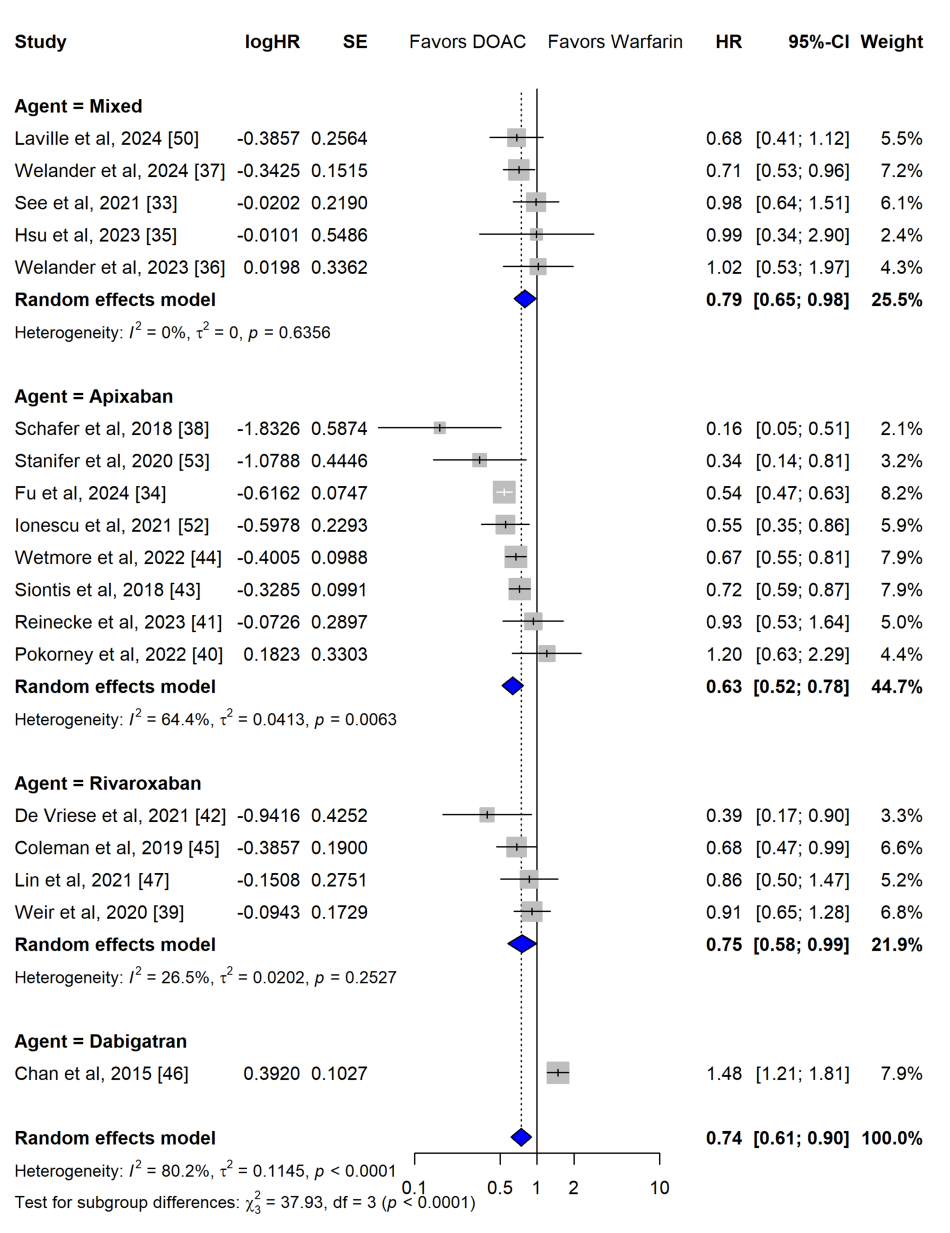
Primary safety outcome (major bleeding) Forest plot for the primary safety outcome, including subgroup analysis by the direct oral anticoagulants (DOAC) agent (apixaban, rivaroxaban, dabigatran, mixed) and calculated prediction interval.

The risk of major bleeding was analysed using data from 18 studies. The random-effects model demonstrated that DOAC therapy significantly reduced the hazard of major bleeding by 26% compared to warfarin (HR, 0.74; 95% CI, 0.61 to 0.90; z = -3.01; p = 0.0026) (Figure 7).

However, this safety finding was characterized by substantial statistical heterogeneity (I^2^ = 80.2%, 95% CI: 69.5-87.1%; Cochran’s Q = 85.90, p < 0.0001; τ^2^ = 0.1145). The calculated prediction interval for major bleeding ranged from 0.35 to 1.56, indicating significant uncertainty regarding the expected effect of DOACs on major bleeding in future individual trials or clinical cohorts.

Subgroup and Meta-Regression Analyses

To explore the sources of the observed heterogeneity, prespecified subgroup analyses and random-effects meta-regressions (REML method) were conducted.

Effect of DOAC Agents on Safety

A subgroup analysis stratified by individual DOAC agents revealed significant differences in the risk of major bleeding (test for subgroup differences: Q = 37.93, df = 3, p < 0.0001). Apixaban demonstrated a robust and statistically significant reduction in major bleeding risk compared to warfarin (HR, 0.63; 95% CI, 0.52-0.78; I^2^ = 64.4%). Rivaroxaban also showed a significant, although slightly attenuated, safety benefit (HR, 0.75; 95% CI, 0.58-0.99; I^2^ = 26.5%). The single study evaluating dabigatran indicated a significantly higher risk of major bleeding compared to warfarin (HR, 1.48; 95% CI, 1.21-1.81). Studies reporting mixed or pooled DOAC cohorts showed a non-significant trend toward reduced bleeding (HR, 0.79; 95% CI, 0.65-0.98; I^2^ = 0.0%). The residual heterogeneity observed within the apixaban subgroup (I² = 64.4%) is multifactorial; exploratory assessments suggest it is driven by variations in dosing regimens (e.g. standard 5 mg versus reduced 2.5 mg twice daily) and differing proportions of dialysis-dependent versus non-dialysis advanced CKD patients across the included studies."

**Figure 8:**
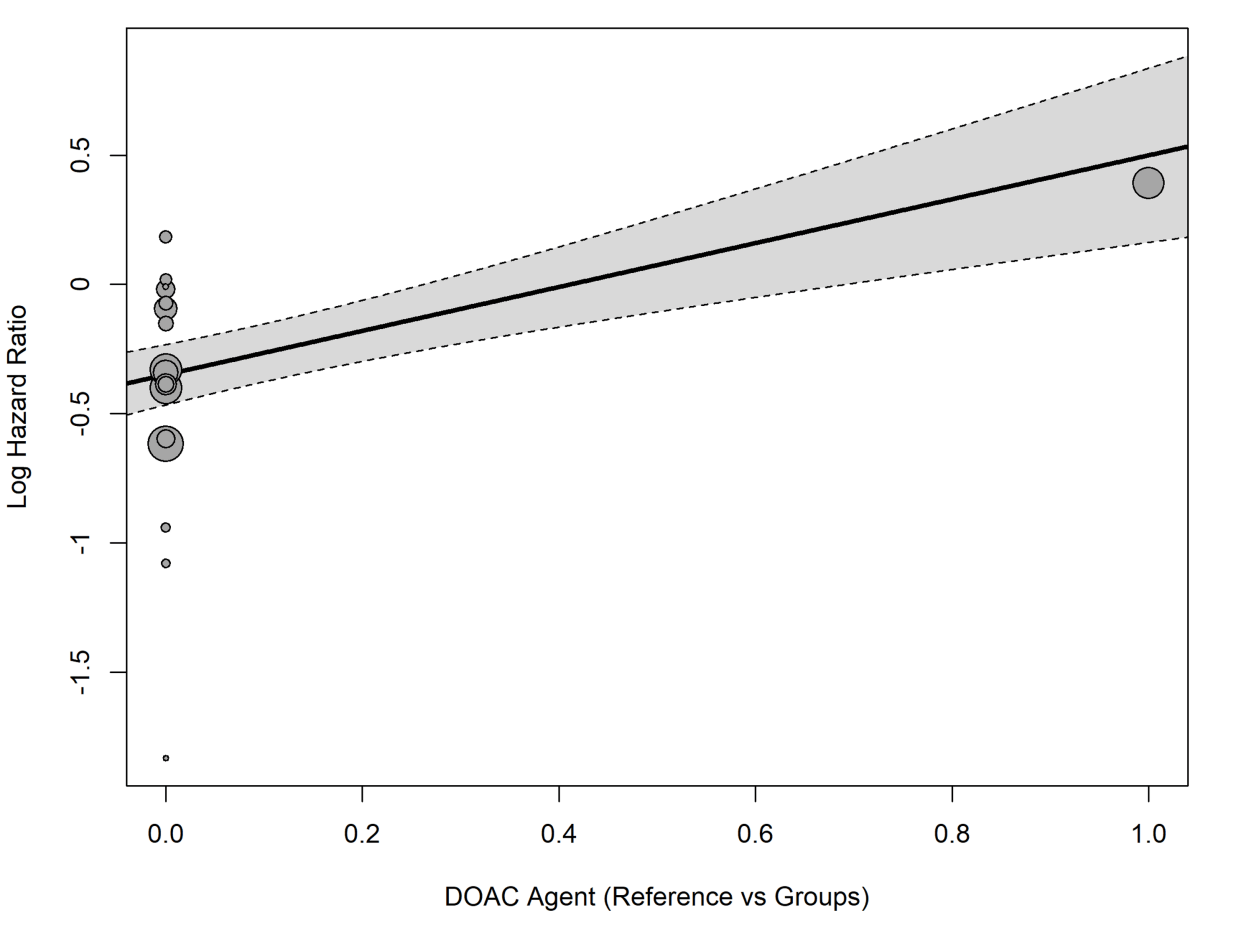
Meta-regression (bubble plot) demonstrating the modifying effect of specific DOAC agents on the log hazard ratio for major bleeding. The x-axis represents the dummy-coded categorical moderator generated by the meta-regression model, where 0.0 represents the reference group and 1.0 represents the specific direct oral anticoagulant (DOAC) agent being evaluated.

Meta-regression confirmed that the specific DOAC agent was a highly significant moderator of bleeding risk (Q_M_ = 22.88, p < 0.0001). The model accounted for 81.80% of the between-study variance (R2 = 81.80%). Specifically, the use of dabigatran significantly increased the log hazard ratio for bleeding relative to the baseline intercept (estimate: 0.8493, SE = 0.1790, p < 0.0001) (Figure 8).

Effect of CKD Stage on Safety

Subgrouping by baseline renal status and comparing dialysis-dependent patients to those with non-dialysis advanced CKD (stages 4-5) did not reveal a statistically significant interaction (test for subgroup differences: Q = 0.72, df = 1, p = 0.3968). DOAC use was associated with reduced bleeding risk in both the non-dialysis advanced CKD (HR, 0.66; 95% CI, 0.49-0.88; I^2^ = 65.2%) and dialysis-dependent cohorts (HR, 0.77; 95% CI, 0.61-0.98; I^2^ = 78.8%).

Effect of Study Design on Efficacy and Safety

When evaluating the primary efficacy endpoint (stroke/SE), subgrouping by study design indicated a non-significant interaction between observational studies and RCTs (Q = 2.52, df = 1, p = 0.1126). However, the magnitude of benefit appeared larger in the RCT subgroup (HR, 0.51; 95% CI, 0.33-0.81; I^2^ = 12.2%) than in the observational studies (HR, 0.76; 95% CI, 0.64-0.92; I^2^ = 39.3%) (Figure 6).

**Figure 9:**
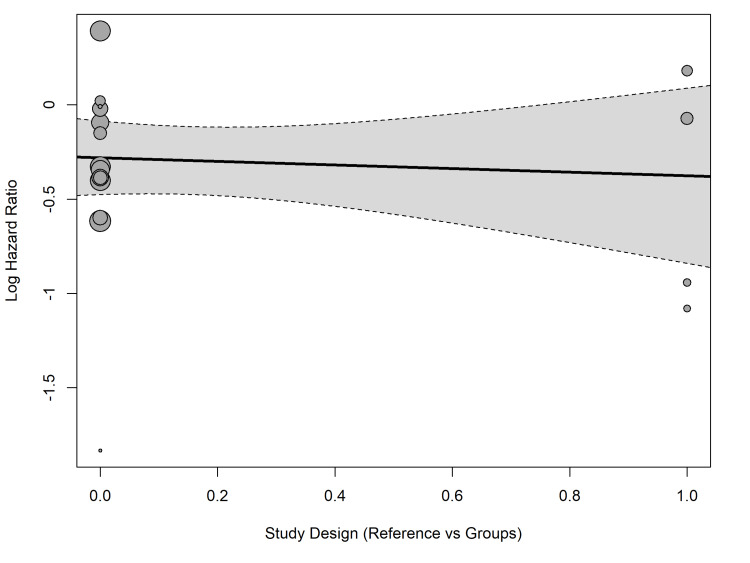
Meta-regression (bubble plot) assessing the impact of study design (RCT vs. observational) on the log hazard ratio for major bleeding.

Meta-regression assessing the impact of study design on the safety outcome (major bleeding) confirmed that study design (RCT vs. observational) was not a significant predictor of the effect size (Q_M_ = 0.1377, p = 0.7105; R^2^ = 0.00%) (Figure 9).

Sensitivity Analysis

**Figure 10:**
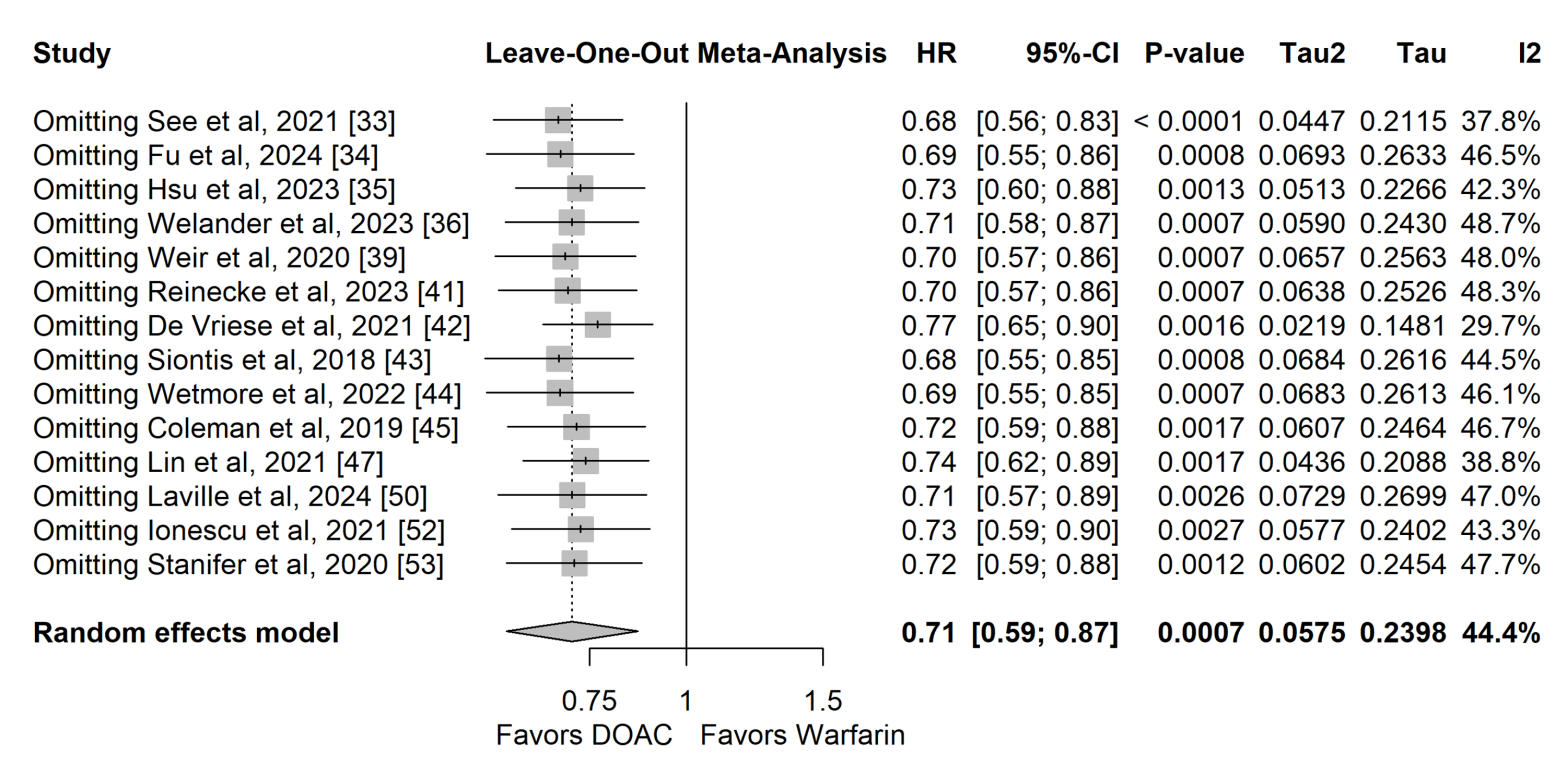
Leave-one-out sensitivity analysis for the primary efficacy outcome (stroke or systemic embolism).

**Figure 11:**
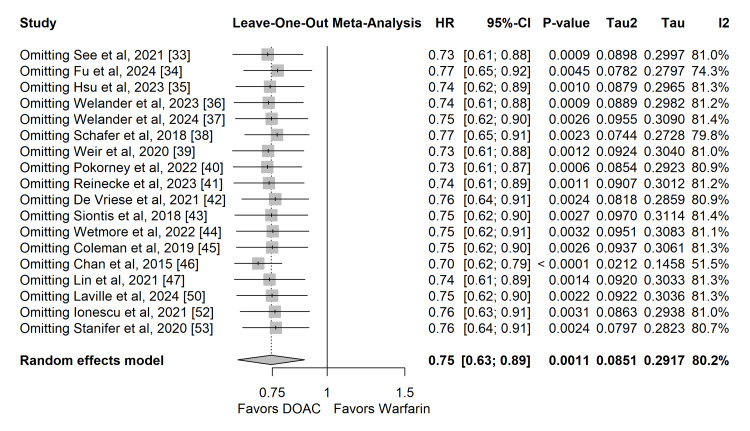
Leave-one-out sensitivity analysis for the primary safety outcome (major bleeding).

A leave-one-out sensitivity analysis was performed to evaluate the robustness of the primary safety and efficacy findings. The systematic removal of individual studies did not meaningfully alter the overall pooled effect sizes or statistical significance for either stroke/SE or major bleeding, indicating that no single study disproportionately drove the results of the meta-analyses (Figures 10, 11). Furthermore, a restricted sensitivity analysis excluding observational studies rated at a serious risk of bias (12.5%) yielded consistent, statistically significant risk reductions for both stroke/SE and major bleeding, confirming that these studies did not disproportionately skew the overall conclusions.

Publication Bias

**Figure 12:**
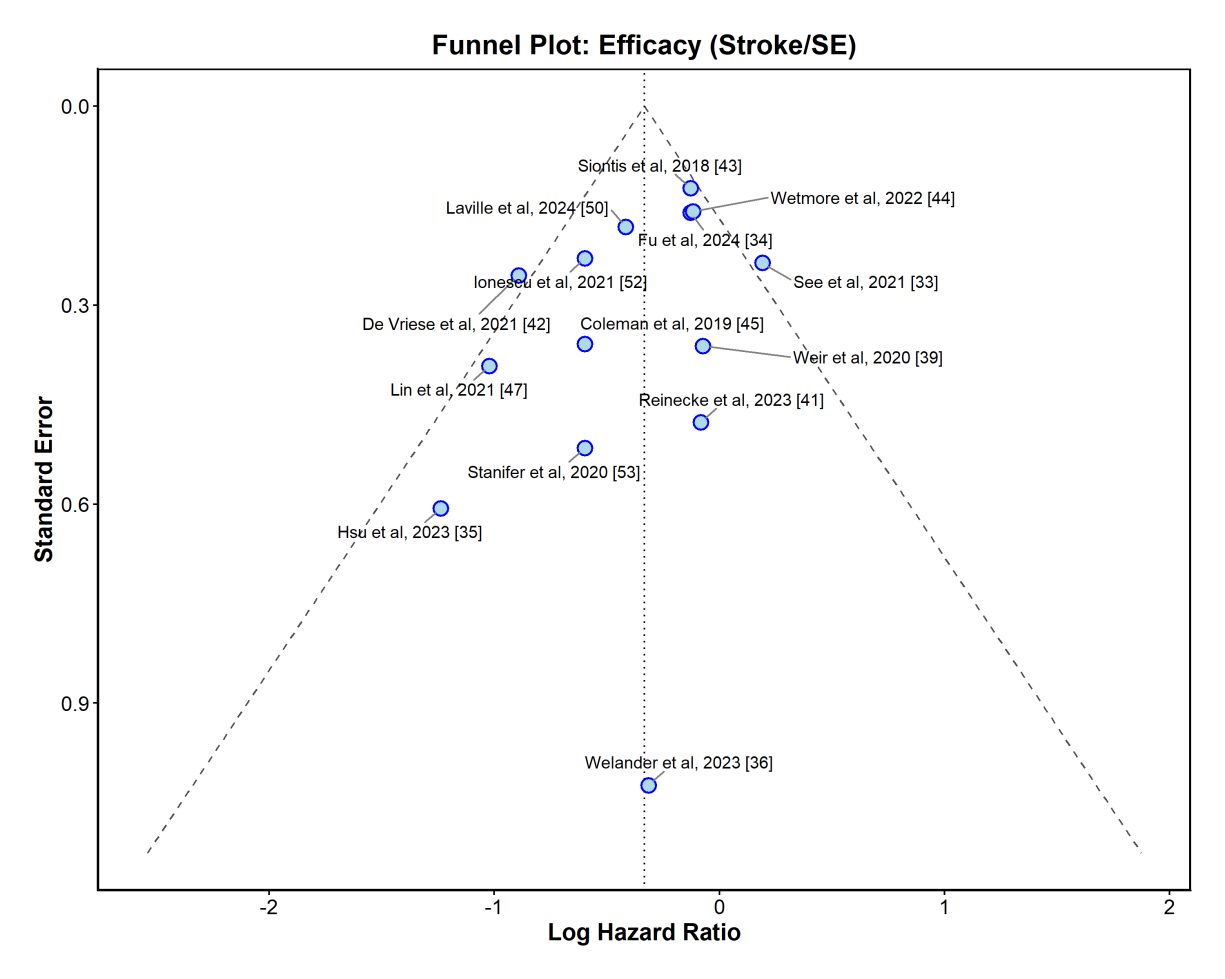
Funnel plot assessing publication bias for the primary efficacy outcome (stroke or systemic embolism).

**Figure 13:**
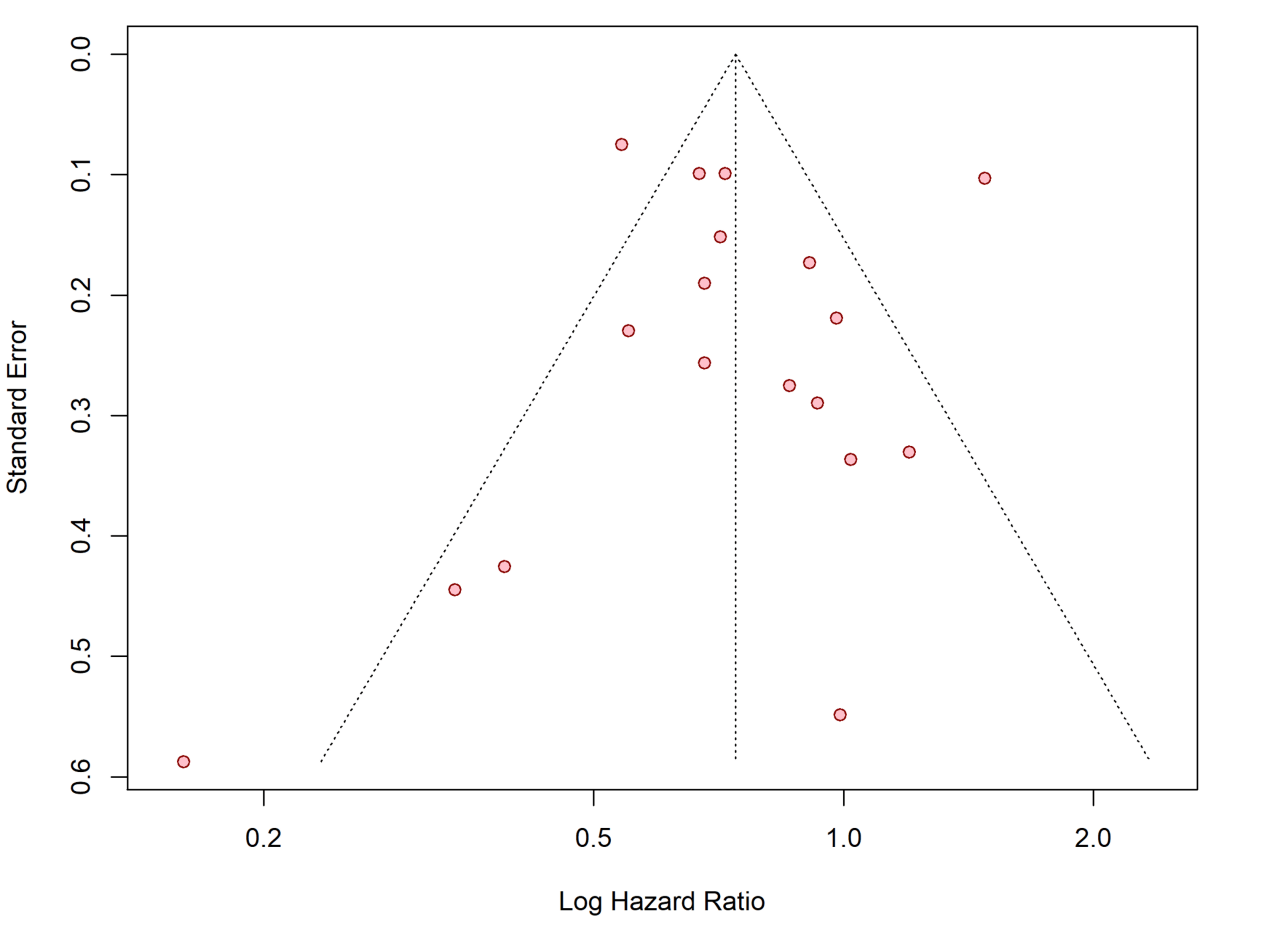
Funnel plot assessing publication bias for the primary safety outcome (major bleeding).

Visual inspection of the funnel plots for both efficacy (stroke/SE) and safety (major bleeding) outcomes suggested a relatively symmetrical distribution of studies around the pooled effect estimates (Figures 12, 13).

This visual assessment was corroborated statistically. For the stroke/SE outcome, Egger’s linear regression test indicated no significant asymmetry (t = − 1.80, df = 12, p = 0.0977), and Begg’s rank correlation test was also not significant (z = − 1.59, p = 0.1124). Similarly, for the major bleeding outcome, neither Egger’s test (t = − 0.01, df = 16, p = 0.9908) nor Begg’s test (z = − 0.27, p = 0.7909) provided evidence of small-study effects or publication bias.

Trial Sequential Analysis (TSA)

**Figure 14:**
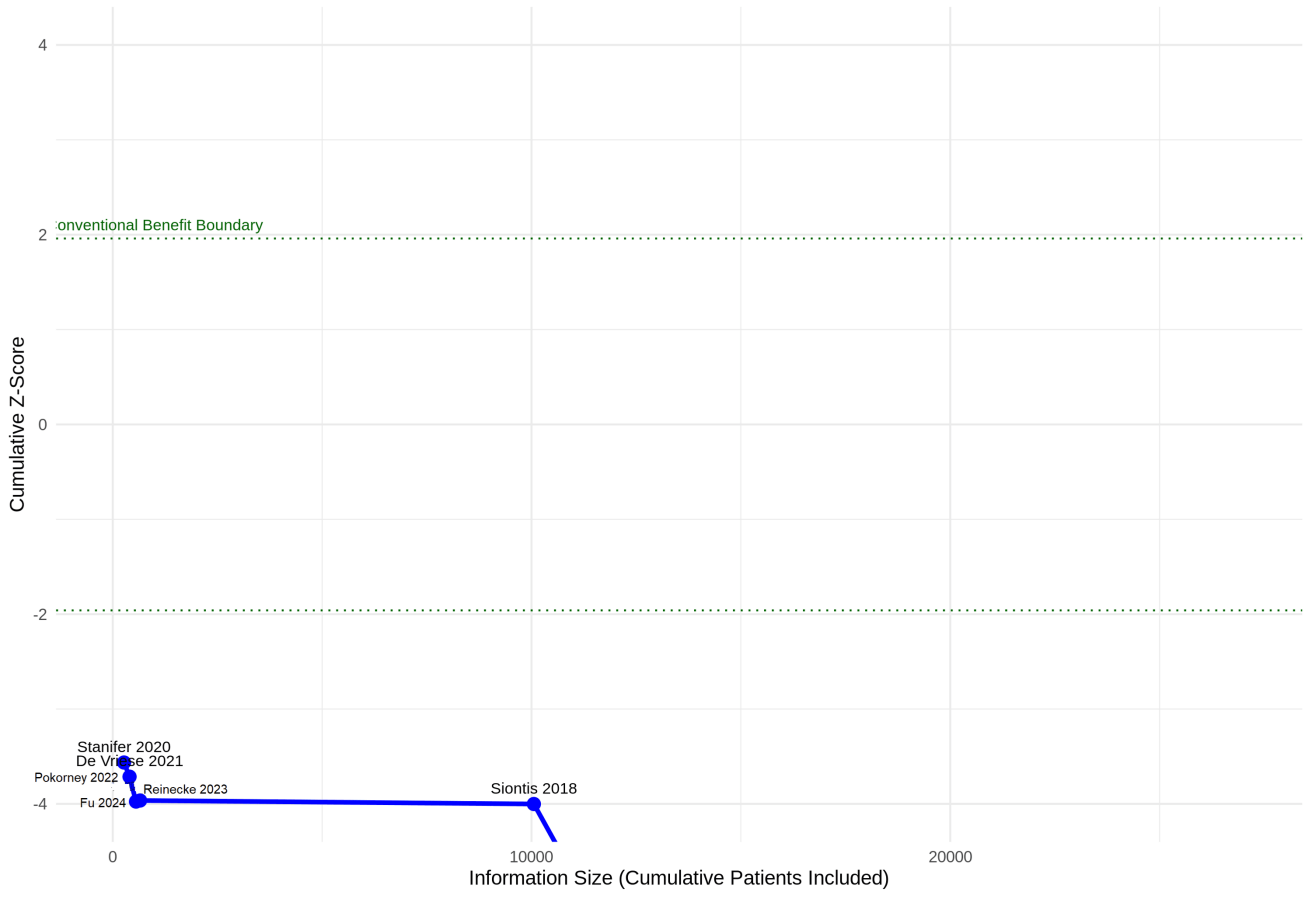
Trial sequential analysis for stroke prevention (direct oral anticoagulant (DOAC) vs. vitamin K antagonist (VKA)) Trial sequential analysis (TSA) for stroke prevention, displaying the cumulative Z-curve against the conventional benefit boundaries and the heterogeneity-adjusted required information size (RIS). Note the substantial difference between the unadjusted RIS (4,717) and the heterogeneity-adjusted RIS (35,457), highlighting the profound impact of inter-study heterogeneity on evidence conclusiveness.

To assess whether the cumulative evidence for stroke prevention is conclusive, a TSA was performed utilizing data from the six studies reporting raw event counts. Assuming a conventional type I error (α) of 5%, a power of 80%, and an anticipated RRR of 20%, the unadjusted RIS was calculated as 4,717 patients. However, after adjusting for the observed heterogeneity, the heterogeneity-adjusted RIS ballooned to 35,457 patients. The cumulative Z-curve clearly crossed the conventional boundary for benefit (Z = 1.96). However, the cumulative information size fell substantially short of the heterogeneity-adjusted RIS of 35,457 (Figure 14), indicating that while the evidence strongly favours DOACs for stroke prevention, the high degree of clinical and statistical heterogeneity in this specific population means that the total sample size remains insufficient to definitively halt further trials on the subject. Assuming a conventional type I error (α) of 5%, a power of 80%, and an anticipated RRR of 20%. selected as a clinically meaningful threshold that aligns with the established benefits of DOACs in pivotal trials for the general AF population, the unadjusted RIS was calculated as 4,717 patients. The massive inflation from an unadjusted RIS of 4,717 to a heterogeneity-adjusted RIS of 35,457 underscores how the high degree of clinical and methodological variability between studies dilutes the certainty of the overall evidence.

GRADE Findings: Certainty of Evidence

The certainty of evidence for the primary outcomes was appraised using the GRADE approach (Table 4). For the primary efficacy outcome (stroke or SE), the evidence was graded as moderate, reflecting a statistically significant reduction in stroke risk (HR 0.72); however, it was downgraded by one grade due to the inclusion of observational data with inherent selection bias and a wide prediction interval (0.43-1.19) that crosses the line of unity.

**Table 4:** GRADE summary of findings: DOACs vs. warfarin in advanced CKD/dialysis Abbreviations: CI, confidence interval; CKD, chronic kidney disease; DOAC, direct oral anticoagulant; GRADE, Grading of Recommendations Assessment, Development and Evaluation; HR, hazard ratio; RCT, randomized controlled trial

Outcome	Anticipated Absolute Effects (Warfarin)	Anticipated Absolute Effects (DOACs)	Relative Effect (95% CI)	No. of Participants (Studies)	Certainty of Evidence (GRADE)	Reason for Downgrading
Stroke or Systemic Embolism	33 per 1,000	24 per 1,000 (20 to 28)	HR 0.72 (0.60 to 0.86)	31,050 (18 observational, 3 RCTs)	⊕⊕⊕⚆ MODERATE	Downgraded 1 level for imprecision (wide prediction interval of 0.43–1.19 crossing the line of unity).
Major Bleeding	95 per 1,000	65 per 1,000 (45 to 90)	HR 0.74 (0.61 to 0.90)	32,112 (18 observational, 3 RCTs)	⊕⊕⊕⚆ MODERATE	Downgraded 1 level for study design (majority observational data). Upgraded for large effect magnitude.
All-Cause Mortality	245 per 1,000	225 per 1,000 (191 to 264)	HR 0.92 (0.78 to 1.08)	28,450 (12 observational, 2 RCTs)	⊕⊕⚆⚆ LOW	Downgraded 2 levels for study design and significant heterogeneity (I² > 75%).

The certainty of evidence for the primary safety outcome (major bleeding) was rated as low to moderate. Although DOACs demonstrated a significant reduction in bleeding risk (HR 0.74), the evidence was downgraded due to substantial statistical heterogeneity (I^2^ = 80.2%) and a wide prediction interval (0.35-1.56). Subgroup analyses by specific DOAC agents significantly improved the consistency of the safety data, particularly for apixaban, which demonstrated higher certainty within its respective subgroup. All-cause mortality was rated as having low certainty because of inconsistent reporting across studies and significant heterogeneity that was not fully explained by subgrouping or meta-regression.

Discussion

This systematic review and meta-analysis represents a robust synthesis of the evidence regarding the comparative efficacy and safety of DOACs versus warfarin in the highest-risk AF population: those with stage 4-5 CKD or undergoing dialysis. The primary findings indicate that DOACs, specifically apixaban and rivaroxaban, are associated with a 28% reduction in stroke or SE and a 26% reduction in major bleeding events compared to warfarin. These results challenge the reliance on VKAs in patients with advanced renal failure and suggest that DOACs may provide superior net clinical benefit.

The safety advantage of DOACs, particularly factor Xa inhibitors, is a critical finding given the bleeding-thrombosis paradox in advanced renal failure [1,2]. Patients with ESKD suffer from uremic platelet dysfunction and altered vessel wall integrity, which exacerbate the risk of hemorrhagic complications from anticoagulation [4]. In these meta-regression and subgroup analyses, the specific DOAC agent was identified as the most significant moderator of this safety profile. Apixaban, which has the lowest renal clearance (27%) of the approved DOACs, demonstrated the most consistent reduction in major bleeding [5,43]. This is pharmacokinetically plausible, as reduced dependence on renal elimination minimizes drug accumulation and the subsequent risk of supratherapeutic anticoagulation [10]. The results regarding dabigatran, although limited to fewer studies, signalled an increased risk of major bleeding, due to its high (80%) renal clearance and poor dialyzability [46]. Dabigatran is currently contraindicated by most regulatory authorities, including the FDA, for patients with a creatinine clearance <30 mL/min. Therefore, our finding of increased bleeding risk with dabigatran confirms known pharmacokinetic limitations and existing guidelines, rather than identifying a novel safety signal. A clear gradient of safety benefit was observed that aligns with the pharmacokinetic reliance on renal clearance. Apixaban (27% renal clearance) demonstrated the most robust safety profile. Rivaroxaban (33% renal clearance) showed a significant but attenuated benefit compared to apixaban, whereas dabigatran (80% renal clearance) was associated with overt harm. Edoxaban (50% renal clearance) could not be separately analyzed due to insufficient dedicated data, reflecting ongoing caution with its use in this population.

The efficacy findings were equally significant. Despite earlier concerns that AF may not be as strong an independent risk factor for stroke in the dialysis population as it is in the general population, this analysis shows a clear benefit for DOACs in stroke prevention. The TSA confirmed that the cumulative evidence has crossed the conventional benefit boundary for stroke reduction. However, the high degree of clinical and statistical heterogeneity in this population meant that the total information size has not yet reached the heterogeneity-adjusted RIS, implying that while the trend is firmly in favour of DOACs, more large-scale, high-quality trials are needed to narrow the confidence intervals and definitively establish the magnitude of benefit in individual clinical subsets (e.g., peritoneal dialysis vs. hemodialysis). These findings are highly consistent with recent meta-analyses, such as those by Kao et al. and AlTurki et al., which also identified apixaban as possessing a superior safety profile in ESRD. However, previous reviews often reported borderline or non-significant efficacy outcomes. By incorporating the most recent data and applying TSA, our updated synthesis demonstrates a clear, statistically significant stroke reduction benefit, thereby advancing the certainty of evidence beyond prior works.

Furthermore, the wide prediction intervals calculated for both efficacy and safety outcomes cross unity, indicating that despite the pooled mean effect strongly favoring DOACs, the true effect in a future study or specific clinical cohort might occasionally favour warfarin, highlighting remaining uncertainty and the need for individualized risk assessment. Although study design was not a statistically significant moderator, the magnitude of stroke reduction appeared numerically more pronounced in the RCT subgroup (HR 0.51) compared to observational studies (HR 0.76). However, this apparent magnified benefit in the RCTs must be interpreted with extreme caution. This discrepancy stems from an overestimation of the true effect driven by the small sample sizes, early termination, and event-driven fragility of the included trials (e.g., RENAL-AF). These RCT estimates lack the statistical power to independently prove superiority, and their wide confidence intervals underscore the danger of overinterpreting these subgroup findings.

A major strength of this study is its comparison of DOACs with well-managed VKA therapy. Previous reviews have been criticized for comparing DOACs to warfarin groups with poor time in therapeutic range (TTR). In the included Swedish and French registries, the mean TTR was approximately 67%, yet DOACs maintained their safety and efficacy advantage [36,50], suggesting that even when VKA therapy is optimally monitored, the pharmacological profile of DOACs offers a safer and more predictable alternative for patients with advanced CKD.

Limitations

Most of the data were derived from observational cohorts, which are subject to unmeasured residual confounding (e.g., frailty, fall risk, and medication adherence) that cannot be fully addressed despite the use of propensity score matching and multivariate adjustments. In addition, the early termination and relatively small sample sizes of the primary RCTs (RENAL-AF and AXADIA-AFNET 8) limit the power of the randomized evidence [40,41]. High statistical heterogeneity was observed in the major bleeding outcome (I² = 80.2%), driven by the varying definitions of major bleeding across the included studies. For instance, while the included RCTs utilized standardized, clinically adjudicated criteria such as the International Society on Thrombosis and Haemostasis (ISTH) definitions, the large observational registries relied on varying ICD-9 and ICD-10 billing codes for hemorrhage. These administrative codes lack cross-registry uniformity and clinical adjudication, introducing a substantial degree of methodological variance that precludes a perfectly standardized comparison of safety events.

In addition, the inclusion of the VALKYRIE trial, which pooled rivaroxaban arms with and without vitamin K2 supplementation, represents a source of clinical heterogeneity, as vitamin K2 could independently influence vascular calcification and long-term thromboembolic outcomes. Reassuringly, however, the leave-one-out sensitivity analyses demonstrated that the exclusion of the VALKYRIE trial did not alter the pooled hazard ratios or statistical significance for either efficacy or safety, confirming that this specific trial did not independently drive the overall conclusions. Finally, explicit data on peritoneal dialysis patients were rarely reported or were pooled indistinguishably with hemodialysis cohorts in large registries. Consequently, the vast majority of analyzed patients were on hemodialysis, severely limiting the generalizability of our findings to the peritoneal dialysis subgroup.

## Conclusions

In patients with non-valvular AF and advanced CKD or dialysis, current evidence suggests the use of factor Xa inhibitors, specifically apixaban and rivaroxaban, may offer a favorable safety and effectiveness profile compared to warfarin. However, these findings must be interpreted with caution due to the reliance on observational data, the potential for unmeasured confounding, and substantial inter-study heterogeneity. A definitive, practice-changing shift away from VKAs in the ESKD population cannot be unequivocally recommended based on this data alone. Large-scale, adequately powered randomized controlled trials are required to confirm these observational trends, clarify agent-specific safety profiles, and establish optimal dosing strategies before routine DOAC use is universally adopted in this highly complex population.
